# Supplementary material for: Transcriptomic and Proteomic Analysis of Drought Stress Response in Opium Poppy Plants during the First Week of Germination
Source: Plants (Basel). 2021 Sep 10;10(9):1878. doi: 10.3390/plants10091878 (PMC8465278; doi:10.3390/plants10091878)
Supplement: Supplementary file 1 [file plants-10-01878-s001.zip › Supplementary material 4_Detailed protocol of proteomics.pdf]

Supplementary Materials S4 – Detailed protocol of proteomic analyses

Whole plants of opium poppy were homogenised by mortar and pestle in liquid nitrogen. Trizol isolates were directly used for filter-aided sample preparation (FASP) as described elsewhere (Wisniewski et al. 2009) using 0.5 μg of trypsin (sequencing grade; Promega). Resulting peptides were analysed by LC-MS/MS.

LC-MS/MS analyses of all peptides were done using RSLCnano system (UltiMate™ 3000, Thermo Fisher Scientific) connected to Orbitrap Q Exactive HF-X spectrometer (Thermo Fisher Scientific). Prior to LC separation, tryptic digests were online concentrated and desalted using trapping column (Acclaim™ PepMap™ 100 C18, dimensions 300 μm × 5 mm, 5 μm particles; Thermo Fisher Scientific, part number 160454). After washing of trapping column with 0.1% FA, the peptides were eluted in backflush mode (flow 300 $nl.min^{-1}$) from the trapping column onto an analytical column (Acclaim™ PepMap™ 100 C18, 3 μm particles, 75 μm × 500 mm; Thermo Fisher Scientific, part number 164570) by 50 min gradient program (3-80% of mobile phase B; mobile phase A: 0.1% FA in water; mobile phase B: 0.1% FA in 80% ACN). Equilibration of the trapping and analytical column was done prior to sample injection to sample loop. The analytical column outlet was directly connected to the Digital PicoView 550 (New Objective) ion source with sheath gas option and SilicaTip emitter (New Objective; FS360-20-15-N-20-C12) utilization. ABIRD (Active Background Ion Reduction Device, ESI Source Solutions) was installed.

MS data were acquired in a data-dependent strategy selecting up to top 20 precursors based on precursor abundance in the survey scan (*m/z* 350–2,000). The resolution of the survey scan was 120,000 (at *m/z* 200) with a target value of 3×10^6 ions and maximum injection time of 100 ms. MS/MS spectra were acquired with a target value of 50,000 and resolution of 15,000 (at *m/z* 200). The maximum injection time for MS/MS was 50 ms. Dynamic exclusion was enabled for 40 s after one MS/MS spectra acquisition. The isolation window for MS/MS fragmentation was set to 1.2 *m/z*.

For data evaluation, we used MaxQuant software (v1.6.17) (Cox J, Mann M. 2008) with inbuild Andromeda search engine (Cox J et al 2011). Search was done against protein databases of *Papaver somniferum* (41,351 protein sequences, version from 03-11-2020, downloaded from ftp://ftp.uniprot.org/pub/databases/uniprot/current_release/knowledgebase/reference_proteomes/Eukaryota/UP000316621_3469.fasta.gz) and cRAP contaminants (112 sequences, version from 22-11-2018, downloaded from http://www.thegpm.org/crap). Modifications were set as

follows for database search: oxidation (M), deamidation (N, Q), and acetylation (Protein N-term) as variable modifications, with carbamidomethylation (C) as a fixed modification. Enzyme specificity was tryptic with two permissible miscleavages. Only peptides and proteins with false discovery rate threshold under 0.01 were considered. Relative protein abundance was assessed using protein intensities calculated by MaxQuant. Intensities of reported proteins were further evaluated using software container environment (https://github.com/OmicsWorkflows/KNIME_docker_vnc; version 4.1.3a). Processing workflow is available upon request: it covers reverse hits and contaminant protein groups (cRAP) removal, protein group intensities log2 transformation and normalization (loessF) and LIMMA statistical tests. For the purpose of this article, protein groups reported by MaxQuant are referred to as proteins.

Mass spectrometry proteomics data were deposited to the ProteomeXchange Consortium via PRIDE (Perez-Riverol et al 2019) partner repository under dataset identifier PXD027435.

References:

Wiśniewski JR, Zougman A, Nagaraj N, Mann M. Universal sample preparation method for proteome analysis. Nat Methods. 2009; 6: 359–362. https://doi.org/10.1038/nmeth.1322

Cox J, Mann M. MaxQuant enables high peptide identification rates, individualized p.p.b.-range mass accuracies and proteome-wide protein quantification. Nat Biotechnol. 2008; 26: 1367–1372. https://doi.org/10.1038/nbt.1511

Cox J, Neuhauser N, Michalski A, Scheltema RA, Olsen J V., Mann M. Andromeda: A Peptide Search Engine Integrated into the MaxQuant Environment. J Proteome Res. 2011; 10: 1794–1805. https://doi.org/10.1021/pr101065j

Perez-Riverol Y, Csordas A, Bai J, Bernal-Llinares M, Hewapathirana S, Kundu DJ, et al. The PRIDE database and related tools and resources in 2019: improving support for quantification data. Nucleic Acids Res. 2019; 47: D442–D450. https://doi.org/10.1093/nar/gky1106
